# Supplementary material for: Prevalence of erectile dysfunction in Thai scleroderma patients and associated factors
Source: PLoS One. 2023 Jan 19;18(1):e0279087. doi: 10.1371/journal.pone.0279087 (PMC9851520; doi:10.1371/journal.pone.0279087)
Supplement: Attachment — Submitted filename: response letter to reviewers_11-8-22.docx [file pone.0279087.s002.docx]

Dear Dr.Luca Navarini,
Academic Editor PLOS ONE

RE: PONE-D-21-36106

Title: Prevalence of erectile dysfunction in Thai scleroderma patients and associated factors

Thank you very much for your letter dated June 15, 2022, including the reviewers’ comments. We are grateful for the opportunity to improve our original manuscript, and for your consideration of this revised version. We have made changes to the original manuscript, addressing the reviewers’ concerns. The changes are listed below. We believe that the changes suggested by the reviewers strengthen the study, making for a greater impact on the field of scleroderma.

Thank you very much for your consideration of this updated version.

Sincerely,

Chingching Foocharoen, MD (on behalf of all the authors)

**Journal Requirements:**

When submitting your revision, we need you to address these additional requirements.

1. Please ensure that your manuscript meets PLOS ONE's style requirements, including those for file naming. The PLOS ONE style templates can be found at
   <https://journals.plos.org/plosone/s/file?id=wjVg/PLOSOne_formatting_sample_main_body.pdf> and 
   <https://journals.plos.org/plosone/s/file?id=ba62/PLOSOne_formatting_sample_title_authors_affiliations.pdf>

**Response:** Our manuscript meets PLOS ONE’s style requirements

2. We note that the grant information you provided in the ‘Funding Information’ and ‘Financial Disclosure’ sections do not match.

When you resubmit, please ensure that you provide the correct grant numbers for the awards you received for your study in the ‘Funding Information’ section.

**Response:** We have followed the matching the funding information and financial disclosure as per the “Funding Information” section.

3. Thank you for stating the following in the Acknowledgments Section of your manuscript: "The authors thank (a) the Research and Graduate Studies, the Scleroderma Research Group and the Faculty of Medicine, Khon Kaen University, for their support, and (b) Mr. Bryan Roderick Hamman for assistance with the English-language presentation under the aegis of the Publication Clinic Khon Kaen University, Thailand."

We note that you have provided funding information that is not currently declared in your Funding Statement. However, funding information should not appear in the Acknowledgments section or other areas of your manuscript. We will only publish funding information present in the Funding Statement section of the online submission form.

Please remove any funding-related text from the manuscript and let us know how you would like to update your Funding Statement. Currently, your Funding Statement reads as follows: "Unfunded studies"

Please include your amended statements within your cover letter; we will change the online submission form on your behalf.

**Response:** The Acknowledgement has been revised according to the format of the journal and the funding statement has been updated.

4. Thank you for stating the following in your Competing Interests section: "No authors have competing interests"

Please complete your Competing Interests on the online submission form to state any Competing Interests. If you have no competing interests, please state "The authors have declared that no competing interests exist.", as detailed online in our guide for authors at <http://journals.plos.org/plosone/s/submit-now> This information should be included in your cover letter; we will change the online submission form on your behalf.

**Response:** The statement of competing interests was added in the cover letter.

5. In your Data Availability statement, you have not specified where the minimal data set underlying the results described in your manuscript can be found. PLOS defines a study's minimal data set as the underlying data used to reach the conclusions drawn in the manuscript and any additional data required to replicate the reported study findings in their entirety. All PLOS journals require that the minimal data set be made fully available. For more information about our data policy, please see <http://journals.plos.org/plosone/s/data-availability>.

Upon re-submitting your revised manuscript, please upload your study’s minimal underlying data set as either Supporting Information files or to a stable, public repository and include the relevant URLs, DOIs, or accession numbers within your revised cover letter. For a list of acceptable repositories, please see <http://journals.plos.org/plosone/s/data-availability#loc-recommended-repositories>. Any potentially identifying patient information must be fully anonymized.

Important: If there are ethical or legal restrictions to sharing your data publicly, please explain these restrictions in detail. Please see our guidelines for more information on what we consider unacceptable restrictions to publicly sharing data: <http://journals.plos.org/plosone/s/data-availability#loc-unacceptable-data-access-restrictions>. Note that it is not acceptable for the authors to be the sole named individuals responsible for ensuring data access.

We will update your Data Availability statement to reflect the information you provide in your cover letter.

**Response:** The Data Availability statement has been updated and the dataset added to supplementary file.

6. Please include your full ethics statement in the ‘Methods’ section of your manuscript file. In your statement, please include the full name of the IRB or ethics committee who approved or waived your study, as well as whether or not you obtained informed written or verbal consent. If consent was waived for your study, please include this information in your statement as well.

**Response:** The ethical issues consideration has been updated in the revised manuscript.

7. Please review your reference list to ensure that it is complete and correct. If you have cited papers that have been retracted, please include the rationale for doing so in the manuscript text, or remove these references and replace them with relevant current references. Any changes to the reference list should be mentioned in the rebuttal letter that accompanies your revised manuscript. If you need to cite a retracted article, indicate the article’s retracted status in the References list and also include a citation and full reference for the retraction notice.

[Note: HTML markup is below. Please do not edit.]

**Response:** We reviewed our references to ensure that they are complete and correct.

**Review Comments to the Author**

Please use the space provided to explain your answers to the questions above. You may also include additional comments for the author, including concerns about dual publication, research ethics, or publication ethics. (Please upload your review as an attachment if it exceeds 20,000 characters)

**Reviewer #1:** This is an interesting and well written study on “Prevalence of erectile dysfunction in Thai scleroderma patients and associated factors”. The authors suggest for the first time to study abnormal urological disease like phimosis, etc.

My minor points are:
Line 196, “A previous study 196 revealed the association between ED and disease severity in terms of extensive skin tightness 197 evaluated by mRSS, having restrictive lung function, high pulmonary arterial pressure, and renal or muscle involvement.” What is the renal involvement? Was renal scleroderma crisis related to ED?

**Response:** Yes, renal involvement was renal crisis. We revised the sentence to make it clearer.

The severity of SSc disease is associated with the risk of developing ED. A previous study revealed the association between ED and disease severity in terms of extensive skin tightness evaluated by mRSS, and having restrictive lung function, high pulmonary arterial pressure, and renal crisis or muscle involvement.^4^

Line 214: “The findings represent the role of vasculopathy for ED development in SSc” Please add the reference Rosato et al and discuss the role of endothelium dysfunction with microvascular damage in SSc patients with ED. (Rosato E, Barbano B, Gigante A, Aversa A, Cianci R, Molinaro I, Quarta S, Pisarri S, Afeltra A, Salsano F. Erectile dysfunction, endothelium dysfunction, and microvascular damage in patients with systemic sclerosis. J Sex Med. 2013 May;10(5):1380-8. doi: 10.1111/jsm.12110. Epub 2013 Feb 27. PMID: 23444914.)

**Response:** Thank you for the suggestion. We reviewed the article and cited the article in the text as below.

Rosato et al. found a positive correlation between the IIEF-5 score and digital sphygmic waves and skin perfusion evaluated using digital photoplethysmography. The authors also found an association between the IIEF-5 score and laser Doppler perfusion imaging of the skin and the digits.^22^ In addition, female sexual dysfunction was reported, and there was a negative correlation between the Doppler indices of the clitoral artery and the Female Sexual Function Index score.^23^ The findings exemplify the role of vasculopathy for ED development in male and female sexual dysfunction in SSc.

**Reviewer #2:** The authors presented a well detailed and thorough assessment of sexual disfunction in male patients affected by Systemic Sclerosis, the quality of the study and the combined urological assessment make it very good sound. However, there are some points that may be implemented to make the paper even better in my opinion:
I'm following the line orders of the downloaded reviewer version

101. evaluated should be changed into "were evaluated for" (maybe just a typo)

**Response:** The term has been changed according to the suggestion.

120. I would suggest a more scientific term for "tip of the penis"

**Response:** Thank you for the suggestion. The term has been changed to “glans of penis”

126. "Univariable analysis": I would suggest to name one by one the statistical tests that were used

**Response:** Thank you for the suggestion. The term has been changed to one used in statistical tests.

126. "Odds ratio": odds ratios for continuous predictors were estimated with a one-predictor logistic regression, exponentiating the coefficient on the predictor and its confidence bounds, and then reporting this as the odds ratio and its 95% confidence interval?

**Response:** Yes, the odds ratio, used for continuous data, was calculated by exponentiation of the coefficient from logistic regression.

I feel this part overall could be more detailed
162-164: I think that this particular data is redundant and suffers from multicollinearity, I would note it down straight afterwards or in the limitations of the study

**Response:** We appreciate the comment. The multicollinearity was not investigated, so we added the statement in limitations of the study as below.

Our study has some limitations: a) we had no control to make a comparison of the prevalence between the general population and SSc patients; b) the study was conducted at a single centre, so the results might not be generalizable; c) there were some missing data such as the WHO functional class; d) there was a small number of patients with no ED that might have resulted in a low power of analysis; and, e) multicollinearity of the data was not investigated.

223-224: the concept about phimosis: How was the skin thightness score in patients with phimosis? Since you said in the results that the skin score for the genitalia in all patients was zero, this assumption might be too arbitrary.

**Response:** Thank you for the comments. Phimosis is tightness of the foreskin of the penis, making the skin unable to be pulled back or retracted. The foreskin in phimosis is not thickness like the skin thickness as in SSc. In addition, skin in the other areas of genitalia (penis and scrotum) were normal, so the skin score of the genitalia were all evaluated with a score of zero, according to the method of mRSS assessment.

Lastly I would cite: J Scleroderma Relat Disord. 2019 Feb;4(1):71-76. doi: 10.1177/2397198318776593.

**Response:** Thank you for the suggestion. We reviewed the article and cited the article in the text as below.

The cause and pathogenesis of ED in SSc remain unclear. Several etiologies contribute to the development of ED, including age, medications, neurogenic, psychogenic, and vasculogenic causes.^2,15–17^ The hypothesis of the combination of fibrosis and vasculopathy in SSc have been thoroughly studied.^18–20^ One study of SSc revealed diminishing systolic and diastolic blood flow velocities (evaluated by duplex ultrasound) in the penile arteries,^19^ and another study compared to normal controls found reduced temperature (as assessed by thermal imaging) of the penis with ED.^20^ Rosato et al. found a positive correlation between the IIEF-5 score and digital sphygmic waves and skin perfusion evaluated using digital photoplethysmography. The authors also found an association between the IIEF-5 score and laser Doppler perfusion imaging of the skin and the digits.^22^ In addition, female sexual dysfunction was reported, and there was a negative correlation between the Doppler indices of the clitoral artery and the Female Sexual Function Index score.^23^ The findings exemplify the role of vasculopathy for ED development in male and female sexual dysfunction in SSc. Tissue fibrosis was a possible pathogenesis of ED in SSc. Nehra et al. found corporal fibrosis in penile tissues obtained during penile prosthesis insertion.^18^ Sex hormone and neurological were thought to be possible roles of ED in SSc, but current data do not support the hypothesis.^19,23^ To date, the cause of ED in SSc has yet to be defined.
